# LncRNA DANCR suppresses acute myocardial infarction in mice via mediating p-RXRA/TRAF2/NIK/IKK/NF-κB signaling pathway

**DOI:** 10.18632/aging.206150

**Published:** 2024-11-14

**Authors:** Li Zhao, Zhi Li

**Affiliations:** 1 Department of Cardiovascular, Affiliated Hospital of Yanan University, Yanan, China

**Keywords:** acute myocardial infarction, LncRNA DANCR, RXRA, TRAF2, the NIK/IKK/NF-κB pathway

## Abstract

Objectives: This study aimed to investigate the role of LncRNA differentiation antagonizing non-protein coding RNA (DANCR) in acute myocardial infarction (AMI).

Methods: A mouse model of AMI was established, and the cardiac contractile function was detected. Next, cardiomyocytes treated with oxygen-glucose deprivation (OGD) were used for gain- and loss-function experiments *in vitro*. RIP analysis was used to verify the binding of DANCR and Retinoid X receptor alpha (RXRA), and Co-IP assay was used to measure the binding of phosphorylated RXRA to TNF receptor associated factor 2 (TRAF2).

Results: The expression of DANCR in myocardial tissues of AMI mice were significantly downregulated. Overexpression of DANCR decreased myocardial infarct size and enhanced cardiac contractile function in AMI mice. Moreover, overexpression of DANCR promoted proliferation and inhibited apoptosis in OGD-induced cardiomyocytes. Mechanism studies demonstrated that DANCR interacted with RXRA and promoted glycogen synthase kinase 3beta (GSK3β)-mediated phosphorylation of RXRA, and phosphorylated RXRA interacted with TRAF2 protein to downregulate TRAF2 protein level. Bexarotene (Bex), an activator of RXRA, inhibited TRAF2 protein expression, while RXRA overexpression had no effect on TRAF2 protein expression. Bex treatment or silencing TRAF2 promoted proliferation and inhibited apoptosis in cardiomyocytes. In addition, silencing DANCR inhibited cardiomyocyte proliferation and induced apoptosis by activating the NIK/IKK/NF-κB pathway, while B022, an inhibitor of NIK, counteracted the effects of DANCR silencing on cardiomyocytes.

Conclusions: Studies demonstrated that DANCR suppressed AMI in mice by mediating p-RXRA/TRAF2/NIK/IKK/NF-κB pathway.

## INTRODUCTION

Acute myocardial infarction (AMI) refers to the acute occlusion of coronary artery, which is one of the most important causes of sudden cardiac death worldwide. Its incidence is mostly sudden, with high mortality, and belongs to the serious type of coronary heart disease [1, 2]. Cardiomyocytes are one of the main cell types involved in cardiac pathological injury, and their regeneration can alleviate myocardial injury by repairing themselves [3]. However, the injury of the heart after AIM cannot be repaired by cardiomyocyte regeneration [4, 5]. During this process, the necrotic myocardium is replaced by a fibrous scar, which triggers cardiac dysfunction and left ventricular remodeling [6]. Apoptosis is a genetically controlled spontaneous cell death process [7]. After AMI, cardiomyocyte apoptosis is an important factor that leads to ventricular function weakening and myocardial remodeling [8, 9]. Therefore, inhibiting cardiomyocyte apoptosis may effectively alleviate the deterioration of AMI.

Long non-coding RNAs (lncRNAs) belong to noncoding RNAs, whose transcripts are approximately 200 nucleotides in length and lack protein coding functions. LncRNAs are involved in the pathogenesis of a variety of diseases, including cancer, liver diseases and myocardial infarction [10, 11]. Reports showed that lncRNA differentiation antagonizing non-protein coding RNA (DANCR) is involved in the development of atherosclerosis [12, 13] and regulate cardiomyocyte apoptosis [14]. However, the role of DANCR in cardiomyocyte injury is controversial. For example, Ruan et al. [14] reported that lncRNA DANCR deletion alleviates cardiomyocyte injury caused by oxygen-glucose deprivation; Qiu et al. [15] reported that DANCR attenuated injury in H9c2 cells induced by hypoxia. However, the role of DANCR in myocardial infarction is not fully understood.

Retinoid X receptor alpha (RXRA) is a nuclear receptor that forms not only homodimers, but also heterodimers with other nuclear receptors [16, 17]. RXRA regulates disease progression by participating in multiple biological processes [17, 18]. In addition, RXRA has been reported that its expression level and functional changes are associated with the progression of a variety of diseases. Previous reports showed that RXRA is closely associated with myocardial infarction and serves as a marker for heart failure [19, 20]. Noteworthy, the functional changes of RXRA caused by phosphorylation modification regulate liver cancer development [18, 21]. N-terminally truncated RXRA (tRXRA) activates the NF-κB pathway by interacting with TRAF2, leading to cancer cell apoptosis, but full-length RXRA has no effect [22]. However, the role of RXRA in AMI remains to be explored.

There are two main branches of NF-κB signaling, called canonical and non-canonical [23]. Available reports shown that the NF-κB pathway was frequently activated in AMI [24, 25]. In addition, a report suggested that the NIK-IKK complex activates the canonical the NF-κB pathway [26]. Based on these reports, we hypothesized that blocking the NIK/IKK/NF-κB pathway may reduce OGD-induced cardiomyocyte apoptosis and improve myocardial infarction in mice. Here, a mouse model of AMI and OGD induced cell model were constructed to investigate the role of DANCR in AMI and verify the above hypothesis.

## MATERIALS AND METHODS

### Animal experiments

Male SD mice (*n* = 32) aged eight weeks were purchased from the Experimental Animal Center of Yanan University (Yanan, China)., individually fed in independent, and maintained in a 12 h light-dark cycle. The animal experimental procedures were approved by the Animal Ethics Committee of the Affiliated Hospital of Yanan University (Approval number: YAU-A-23041). Subsequently, mice were randomly divided into four groups: Sham+LV-NC group, Sham+LV-DANCR group, AIM+LV-NC group, and AIM+LV-DANCR group with 8 mice in each group. We induced Myocardial infarction (MI) by surgical ligation of the left anterior descending coronary artery (LAD). First, the left fourth intercostal space chest were opened and ligated the LAD with a 6–0 silk suture. Mice in the sham group underwent the same surgical procedure without the ligation of LAD. Twenty-four hours after MI surgery, lentivirus carrying DANCR overexpression vector (LV-DANCR) or control (LV-NC) were injected into the apex of the heart of AMI mice. At the end of the 21-day experimental period, cardiac function indexes of all mice were collected, Evans Blue dye (1 mL of a 2.0% solution) was injected into the coronary circulation, and then the hearts were rapidly excised and stained with 2,3,5-triphenyl tetrazolium chloride (TTC). The results of which were taken as the infarct area (INF)/the ischemic region (area at risk, AAR) × 100%.

### Cell culture and treatment

The mouse heart-derived cell line (H9c2) and HEK293T cells were purchased from Wuhan Pricella Biotechnology Co., Ltd. The cells were maintained in DMEM containing 10% FBS (Hyclone, USA) and 1% penicillin-streptomycin at 37°C in a humidified 5% CO_2_ atmosphere. H9c2 cells were pretreated with Bexarotene (Bex, 15 µg/mL) or B022 (10 µg/mL) for 3 h, and then treated with OGD for 0, 2, 6, 12 and 24 h., next lysed the cells, and disrupted them with sonication.

### Cell transfection

The full-length sequence of lncRNA DANCR (pc-DANCR), RXRA (pc-RXRA), and TRAF2 (pc-TRAF2), and the specific short hairpin RNA of DANCR (sh-DANCR) and RXRA (sh-RXRA) were synthesized by GenePharma (Shanghai, China). Cells density reached about 70%, and the vectors were transfected into H9c2 cells by using the Lipofectamine^®^ 3000 reagent (Thermo Fisher Scientific, USA). The sequences used for sh-DANCR are as follows: 5′-GAAUCAGAUCUGCAUGGCUAUGACA-3′, the sequences used for sh-RXRA are as follows: 5′-GCAGCACTGAGGATATCAA-3′, and a non-targeting sequence was used as control (sh-NC).

### Quantitative real-time RT-PCR (RT-qPCR)

Total RNA was isolated with Trizol reagents (Invitrogen, USA) and synthesized First-strand cDNA samples by using a transcript First-Strand cDNA Synthesis Supermix kit (Beijing TransGen Bio, Beijing, China). qPCR was performed with Fast SYBR Green Master Mix (Applied Biosystems, USA) in a Biosystems StepOne^™^ Real-Time qPCR machine (Applied Biosystems, USA). β-actin was used as a control, and the relative RNA expression was calculated by using the 2^−ΔΔCT^ method. The sequences of primers are as follows (5′ to 3′): DANCR forward 5′-AGAAACCCGTGACTGAATGG-3′, and reverse 5′-ACTCACATGGCCCTCACTTC-3′; TRAF2 forward 5′-CCT ACT GCT GAG CTC ATT CT-3′, and reverse 5′-CAA TCT TGT CCT GGT CTA GC; GSK3β forward 5′-GCT GTG TGT TGG CTG AAT TGT-3′, and reverse 5′-CTG CTC CTG GTG AGT CCT TT-3′; RXRA forward 5′-CAA GCA GCA GAC AAG CAG-3′, and reverse 5′-GCC AGG AGA ATC CCA TCT-3′.

### Western blot analysis

RIPA buffer was used to lyse H9c2 cells and tissues, and the protein concentration was detected with a BCA kit (Thermo Fisher Scientific, USA). Next, proteins were separated by using 10% SDS-PAGE and transferred to PVDF membranes. The primary antibodies were used as follows, anti-RXRA (1:600, all form Santa Cruz Bio, USA), anti-p-RXRA (1:600), anti-TRAF2 (1:600), anti-Iκ-Ba (1:600), anti-NF-κB/p52 (1:600), anti-p-NF-κB/p52 (1:600), and anti-β-actin (1:1000, Sigma, USA). HRP-conjugated IgG (1:500, Abcam, USA) were used. The membranes were visualized with an ECL kit, and the band gray values were quantified with the Scion Image software. β-actin was used as a reference.

### Co-immunoprecipitation (Co-IP)

The cells were lysed in lysis buffer, and the lysates were incubated with Anti-RXRA, Anti-p-RXRA, Anti-GSK3β antibodies or IgG chelated glutathione-agarose beads overnight. Next, the proteins attached to the beads were dissociated with elution buffer and detected with Western blot.

### Flow cytometry assays

The apoptosis was measured with a FACS Aria Sorter kit (Becton Dickinson, USA). The cells were re-suspended in 500 µL of buffer solution (PBS containing calcium), and then 5 µL of Annexin V-FITC and 10 µL of propidium iodide (PI) were added to each sample and incubated in the dark for 15 min. Next, 400 µL of binding buffer was added to each sample, and the apoptosis rate was immediately analyzed by using a flow cytometer (Becton Dickinson, USA).

### CCK-8 test

H9c2 cells at a density of 5 × 10^3^ cells/well were seeded into 96-well plates and incubated at 37°C for 72 h, and then 10 µL of CCK-8 solution was added to each well and incubated for another 2 h under the same conditions. Cell viability was calculated according to the absorbance value read at 450 nm.

### Statistical analysis

The data are shown as the mean ± SEM, and data analysis was performed by using SPSS version 20.0 software. One-way ANOVA and two-way ANOVA were used to evaluate the differences between multiple groups, and *t*-test was used to evaluate the statistical differences between two groups. *P* < 0.05 were considered to indicate statistically significant differences.

### Data availability statement

The data and materials used and analyzed during the current study are available from the corresponding author on reasonable request.

## RESULTS

### The expression of DANCR was downregulated in AMI mice

**Figure 1:**
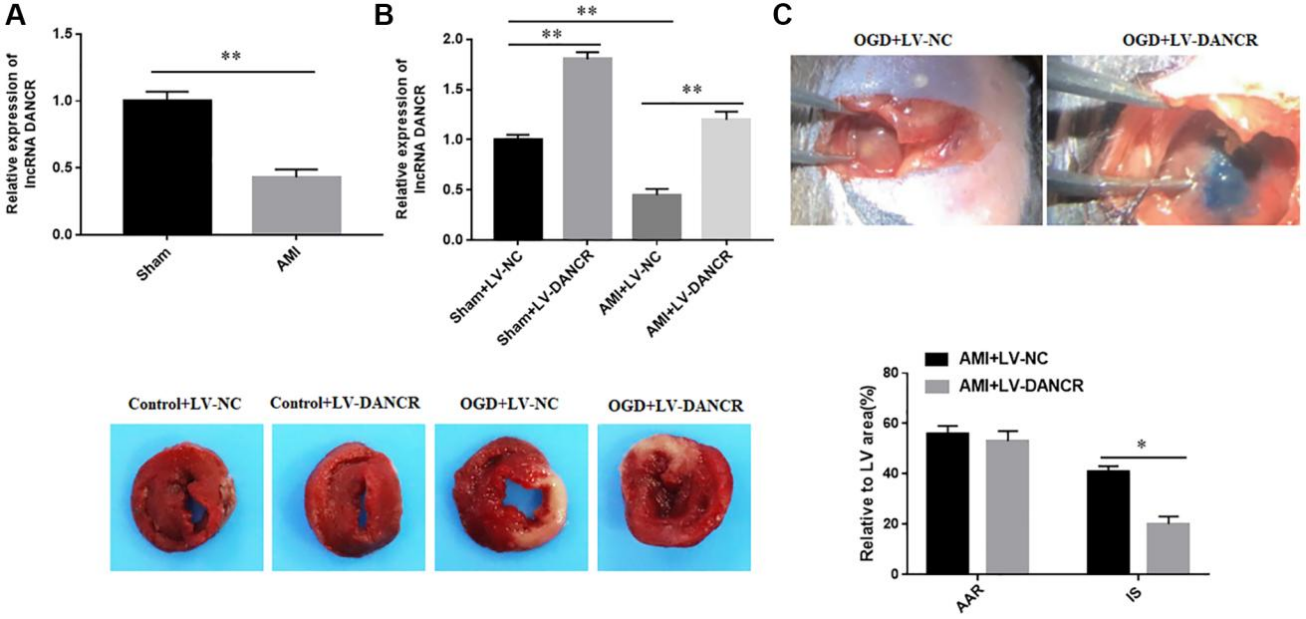
**The expression of DANCR in AMI mice.** (**A**) The expression of DANCR in myocardium of mice in the sham and AMI groups was detected with RT-qPCR. (**B**) Overexpression efficiency of DANCR was detected with RT-qPCR. (**C**) The infarct size (IS) and the areas at risk (AAR) of AMI mice were measured by using Evens blue and TTC staining. ^*^P < 0.05, ^**^P < 0.01.

The results of RT-qPCR assay showed that expression of DANCR was significantly downregulated in the infarcted myocardium of AMI mice (Figure 1A). Next, LV-DANCR was injected into mice, and it was found that DANCR was successfully overexpressed in both AMI mice and normal mice (Figure 1B). Moreover, the effects of DANCR overexpression on the infarct size (IS) and the areas at risk (AAR) of AMI mice were examined by using Evens blue and TTC staining. The results showed that overexpression of DANCR had not significantly changed the AAR in AMI mice, but significantly reduced IS in AMI mice (Figure 1C). These results indicated that overexpression of DANCR reduced IS in AMI mice.

### Overexpression of DANCR improved the cardiac function of AMI mice

**Figure 2:**
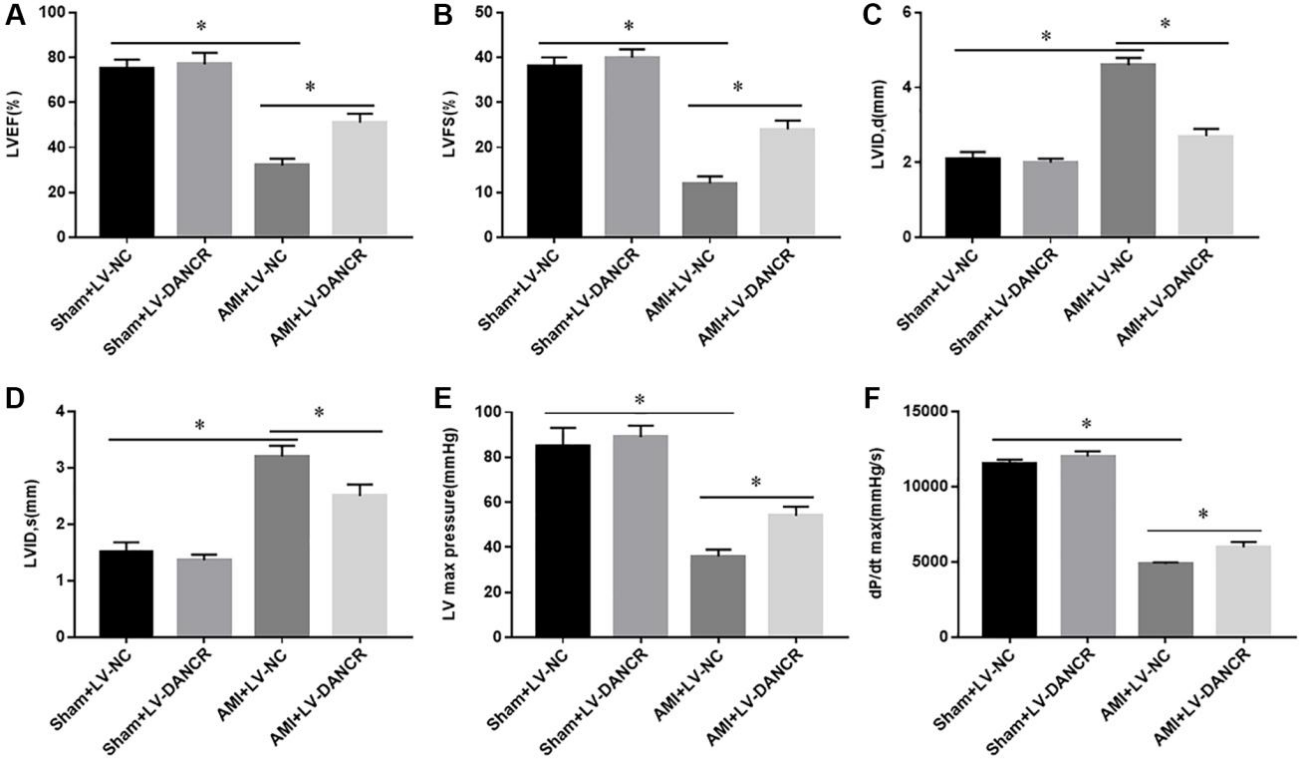
**Effects of DANCR overexpression on the cardiac function of AMI mice.** LV-DANCR and LV-NC were intramyocardially injected into the apex with a 30-G needle 24 h after AMI surgery. And 21 days after AMI, cardiac function of mice was measured by using metrics including (**A**) left ventricular ejection fraction (LVEF), (**B**) left ventricular fractional shortening (LVFS), (**C**, **D**) diastolic and systolic left ventricular internal diameter (LVID,d and LVID,s), (**E**) left ventricular (LV) maximum pressure and (**F**) dP/dt maximum rate. ^*^P < 0.05.

To explore the role of DANCR in AIM, the effects of DANCR overexpression on the recovery of cardiac dysfunction after AMI in mice was assessed. The results showed that left ventricular ejection fraction (LVEF), left ventricular fractional shortening (LVFS), left ventricular (LV) maximum pressure, and dP/dt maximum rate were significantly decreased, and diastolic and systolic left ventricular internal diameters (LVID,d and LVID,s) were markedly elevated in AMI mice. Overexpression of DANCR partially reversed the changes of these indicators in AMI mice, whereas this phenomenon was not observed in normal mice (Figure 2A–2F). These observations indicated that overexpression of DANCR improved the impaired cardiac contractile function in AMI mice.

### Overexpression of DANCR inhibited OGD-induced cardiomyocyte apoptosis

**Figure 3:**
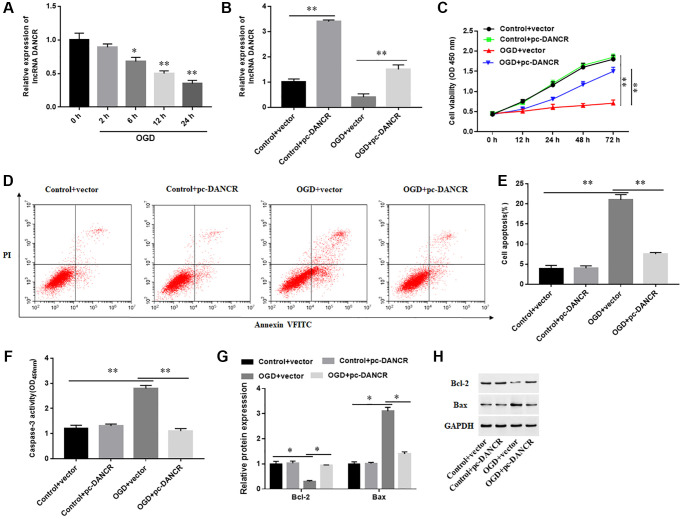
**Effects of DANCR overexpression on the behavior of cardiomyocytes.** (**A**) The expression of DANCR in H9C2 cells treated with OGD for 0, 2, 6, 12 and 24 h. H9C2 cells were transfected with DANCR overexpression vector (pc-DANCR) or control (vector), and then treated with OGD for 24 h. (**B**) The expression of DANCR was detected with RT-qPCR. (**C**) Cell viability was measured by using CCK-8. (**D**, **E**) Cell apoptosis was assessed by using Flow cytometry. (**F**) The activity of caspase-3 was assayed by ELISA. (**G**, **H**) Western blotting was used to detect the protein levels of Bcl-2 and Bax. ^*^P < 0.05, ^**^P < 0.01.

The effect of DANCR overexpression on cardiomyocyte behavior was explored, and the results showed that DANCR expression was markedly downregulated in a time-dependent manner in OGD-induced H9C2 cells (Figure 3A). Overexpression of DANCR sensibly increased the levels of DANCR in both H9C2 cells treated with or without OGD (Figure 3B). Furthermore, OGD induction markedly inhibited cell proliferation, promoted cell apoptosis, enhanced Caspase-3 activity, increased Bax protein levels, and reduced Bcl-2 protein levels, while overexpression of DANCR significantly promoted proliferation and induced apoptosis of H9C2 cells (Figure 3C–3H). The results demonstrated that overexpression of DANCR inhibited OGD-induced cardiomyocyte apoptosis.

### DANCR promoted GSK3β-mediated phosphorylation of RXRA protein in cardiomyocytes

**Figure 4:**
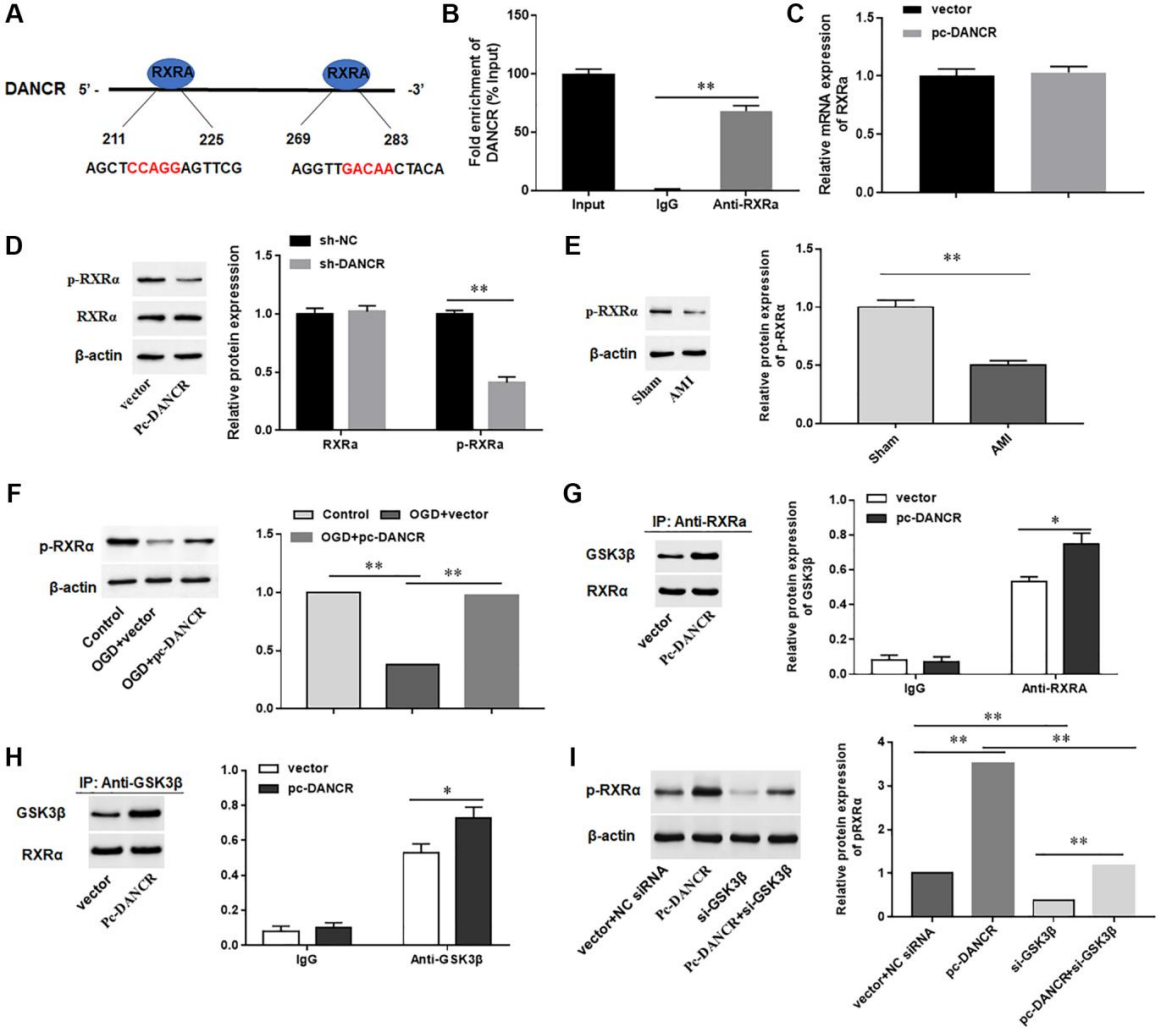
**DANCR promoted GSK3β-mediated phosphorylation of RXRA protein in cardiomyocytes.** (**A**) Schematic diagram of the predicted RXRA binding sites in DANCR. (**B**) RIP assay was used to verify the association of RXRA with DANCR. (**C**) DANCR overexpression vector (pc-DANCR) and control (vector) were transfected into H9C2 cells. The mRNA expression of RXRA was detected with RT-qPCR. (**D**) shRNA of DANCR (sh-DANCR) and control (sh-NC) were transfected into H9C2 cells, and the levels of RXRA total protein and p-RXRA protein were measured by Western blotting. (**E**) The protein expression of p-RXRA in myocardium of mice in the sham and AMI groups was detected with Western blotting. (**F**) H9C2 cells were transfected with DANCR overexpression vector (pc-DANCR) and control (vector), and then treated with OGD. The protein levels of p-RXRA were detected with Western blotting. (**G**) Co-IP analysis was used to detect the interaction between RXRA and GSK3β by using an antibody of Anti-RXRA. (**H**) Co-IP analysis was used to detect the interaction between RXRA and GSK3β by using an antibody of Anti-GSK3β. (**I**) H9C2 cells were transfected with DANCR overexpression vector (pc-DANCR) and/or GSK3β siRNA (si-GSK3β), and then treated with OGD. The protein level of p-RXRA was detected with Western blotting. ^*^P < 0.05, ^**^P < 0.01.

LncRNAs are able to bind functional proteins as sponges and then affect the expression of their downstream genes. Therefore, we hypothesized that DANCR plays a role in AMI through its binding proteins. The binding proteins of DANCR were examined by using a JASPAR database, and RXRA protein was identified as the most potential candidate protein for DANCR binding (Figure 4A). Next, we performed RIP assay and found that DANCR was markedly enriched in RNA-protein complexes (Figure 4B). Moreover, the mRNA level of RXRA in DANCR overexpressing H9C2 cells did not change, while silencing DANCR markedly decreased the level of phosphorylated RXRA, but the total RXRA protein level did not change (Figure 4C, 4D). Meanwhile, we also found a decrease of p-RXRA level in myocardium of AIM mice (Figure 4E). Whereas overexpression of DANCR reversed the downregulation of p-RXRA induced by OGD in H9C2 cells (Figure 4F). The reports shown that since glycogen synthase kinase 3 beta (GSK3β) induces the phosphorylation of RXRA [27, 28], so we examined whether the phosphorylation of RXRA protein regulated by DANCR was dependent on GSK3β. The results showed that overexpression of DANCR significantly promoted the interaction between RXRA and GSK3β in H9c2 cells (Figure 4G, 4H). However, silencing GSK3β inhibited the induction of phosphorylation of RXRA protein by DANCR (Figure 4I). Together, these results suggested that DANCR mediated phosphorylation of RXRA in H9c2 cells is GSK3β dependent.

### Activator of RXRA inhibited OGD-induced cardiomyocyte apoptosis

**Figure 5:**
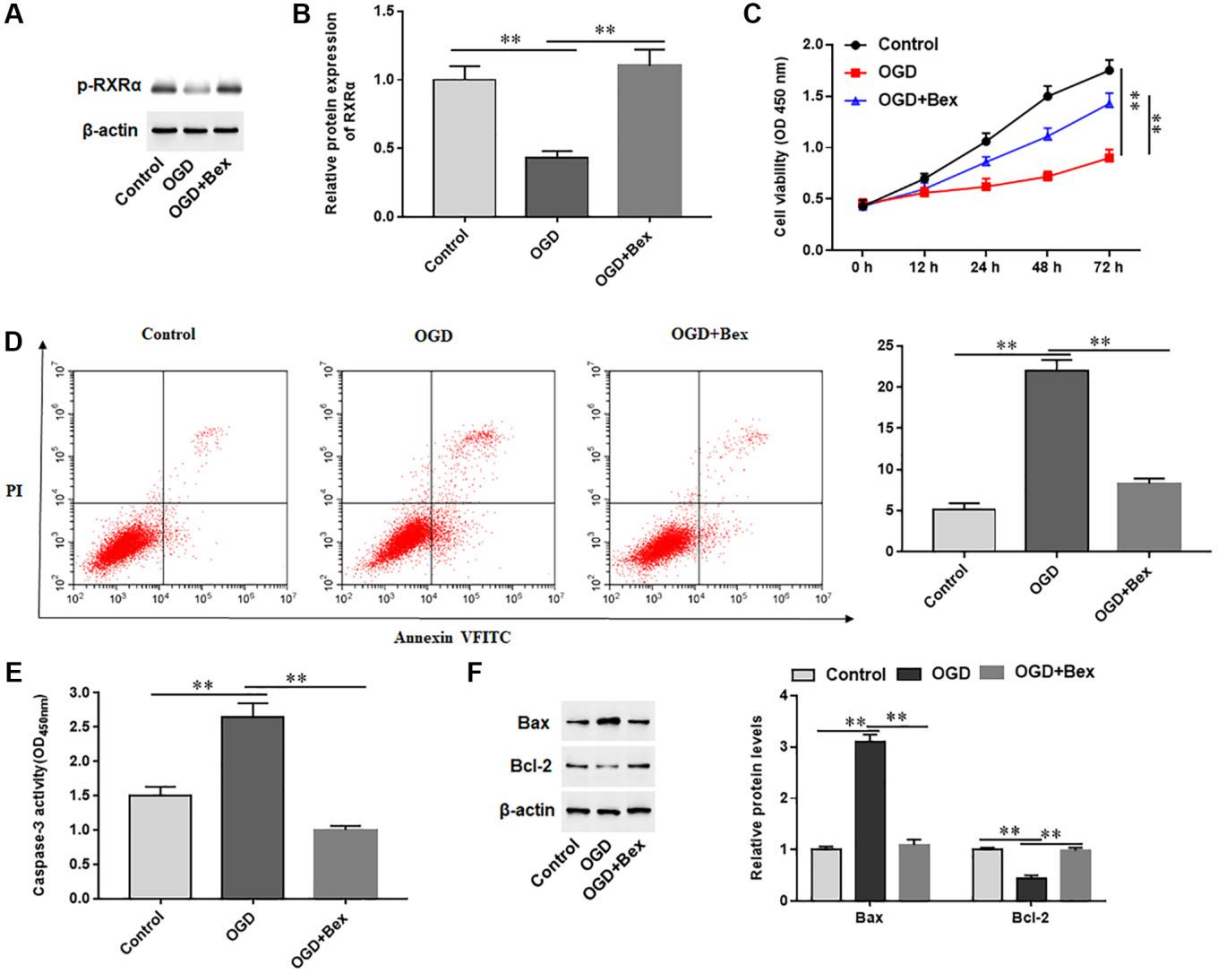
**Effects of p-RXRA on cardiomyocytes treated with OGD.** H9C2 cells were pretreated with Bex and treated with OGD. (**A**, **B**) The protein expression of p-RXRA was detected with Western blotting. (**C**) Cell viability was measured by using CCK-8. (**D**) Cell apoptosis was assessed by using Flow cytometry. (**E**) The activity of caspase-3 was assayed by ELISA. (**F**) Western blotting was used to detect the protein levels of Bcl-2 and Bax. ^*^P < 0.05, ^**^P < 0.01.

To investigate the role of RXRA on cardiomyocytes treated with OGD, we pretreated OGD-induced H9C2 cells with Bex, an activator of RXRA. OGD treatment markedly decreased the level of p-RXRA in H9C2 cells, while Bex treatment increased p-RXRA levels (Figure 5A, 5B). Furthermore, Bex significantly promoted cell proliferation, inhibited cell apoptosis, reduced Caspase-3 activity, decreased Bax protein levels, and increased Bcl-2 protein levels in OGD-treated H9C2 cells (Figure 5C–5F). The results demonstrated that p-RXRA promoted proliferation and inhibited apoptosis in cardiomyocytes induced by OGD.

### Phosphorylated RXRA interacted with TRAF2 to downregulate TRAF2 protein

**Figure 6:**
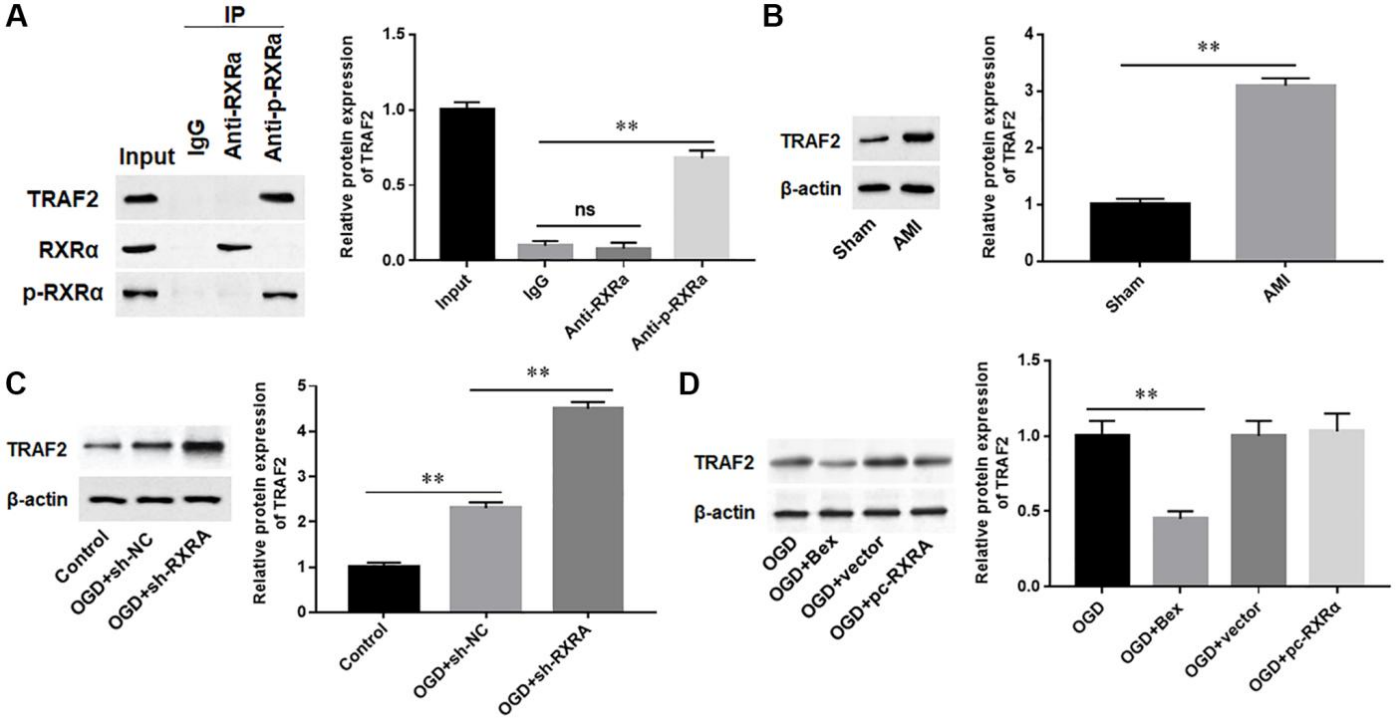
**Phosphorylated RXRA interacted with TRAF2 to downregulate TRAF2 protein.** (**A**) The interaction between RXRA and TRAF2 was detected by Co-IP analysis. (**B**) The protein expression of TRAF2 in mice of the sham and AMI groups was detected with Western blotting. (**C**) H9C2 cells were transfected with sh-RXRA or sh-NC, and then treated with OGD, and the protein expression of TRAF2 was detected with Western blotting. (**D**) H9C2 cells were transfected with RXRA overexpression vector (pc-RXRA) and control (vector), and then treated with OGD and/or Bex. The protein expression of TRAF2 was detected with Western blotting. ^**^P < 0.01.

TRAF2 is a key regulator of cardiac function [29, 30]. Therefore, we speculated that the function of RXRA in cardiomyocytes may be associated with TRAF2. The results of Co-IP assay showed that phosphorylated RXRA protein was able to interact with TRAF2 protein, but the total RXRA protein could hardly bind to TRAF2 protein (Figure 6A). Moreover, we assessed TRAF2 expression levels in AMI mice and found that TRAF2 protein level was significantly upregulated in myocardium of AIM mice (Figure 6B). Meanwhile, we observed that silencing RXRA further promoted the protein expression of TRAF2 in OGD-induced H9C2 cells, while overexpression of RXRA had no effect on TRAF2 protein expression, which was inhibited by Bex, an RXRA phosphorylation activator (Figure 6C, 6D). These data suggested that phosphorylated RXRA inhibited TRAF2 protein expression by binding to TRAF2 protein.

### Overexpression of TRAF2 promoted cardiomyocyte apoptosis

**Figure 7:**
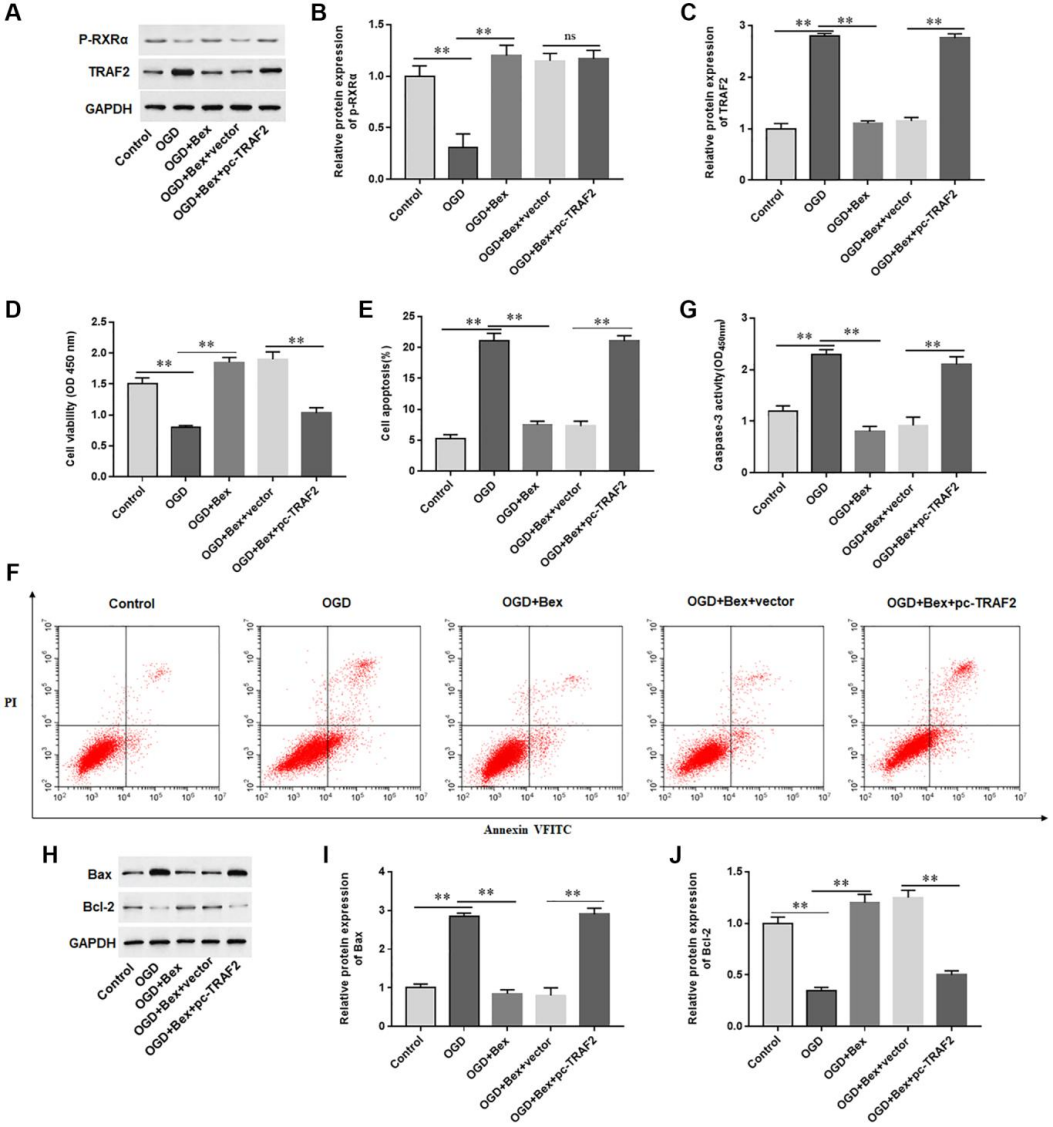
**Overexpression of TRAF2 reversed the protective effect of Bex on cardiomyocytes.** H9C2 cells were transfected with TRAF2 overexpression vector (pc-TRAF2) or control (vector), and then treated with OGD and/or Bex. (**A**–**C**) The protein expression of p-RXRA and TRAF2 was detected with Western blotting. (**D**) Cell viability was measured by using CCK-8. (**E**, **F**) Cell apoptosis was assessed by using Flow cytometry. (**G**) The activity of caspase-3 was assayed by ELISA. (**H**–**J**) Western blotting was used to detect the protein levels of Bcl-2 and Bax. ^*^P < 0.05, ^**^P < 0.01.

The results showed that Bex treatment increased p-RXRA levels and inhibited TRAF2 protein levels in OGD-treated H9C2 cells (Figure 7A–7C). Furthermore, we observed that Bex treatment markedly promoted proliferation, inhibited cell apoptosis and Caspase-3 activity, reduced Bax protein levels, and elevated Bcl-2 protein levels in OGD-induced H9C2 cells, while overexpression of TRAF2 markedly offset the protective effect of Bex on H9C2 cells, (Figure 7D–7J). The results demonstrated that p-RXRA reduced OGD-induced cardiomyocyte apoptosis through inhibiting TRAF2 upregulation.

### Silencing DANCR induced cardiomyocyte apoptosis by activating the NIK/IKK/NF-κB pathway

**Figure 8:**
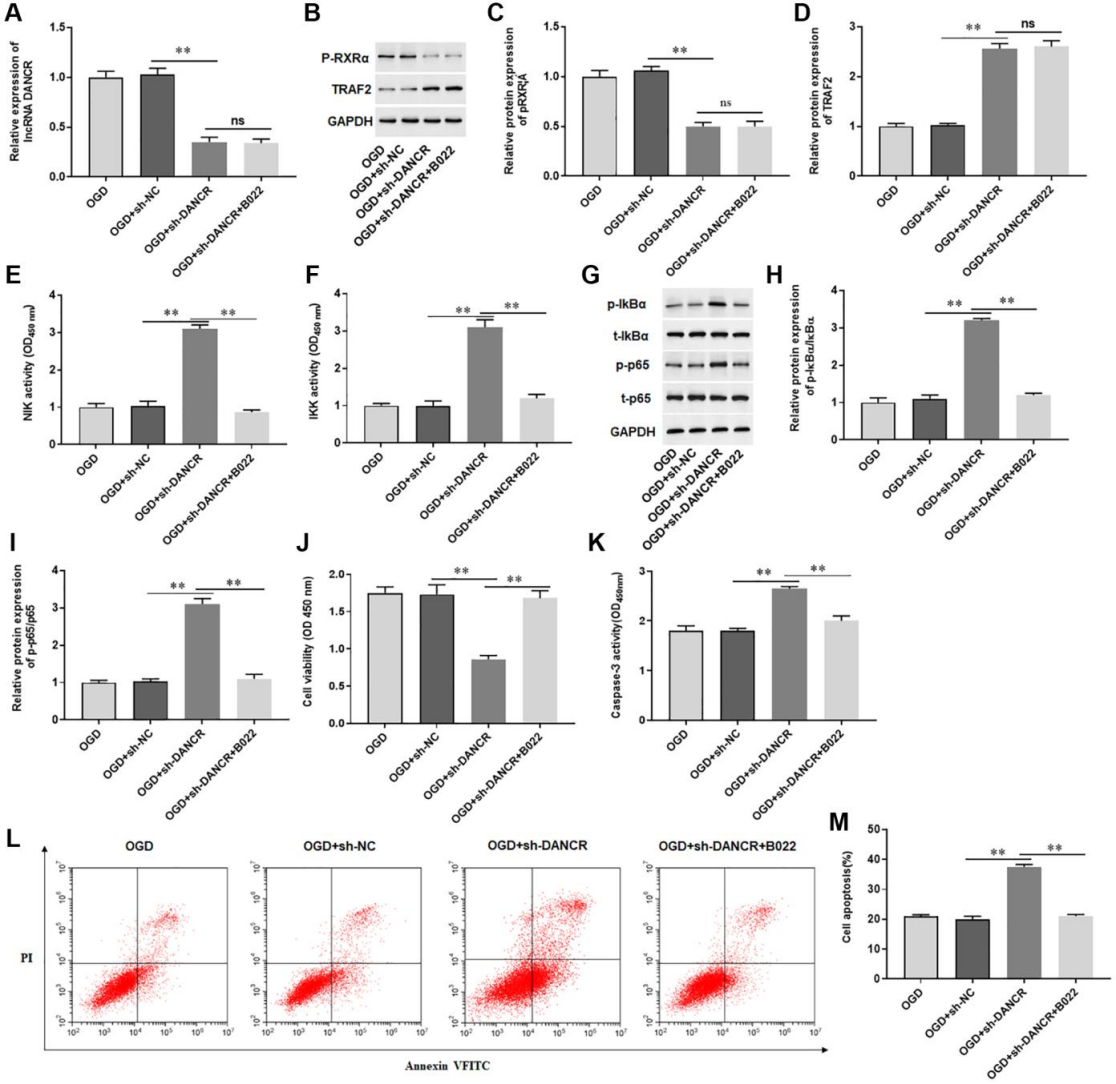
**Silencing DANCR induced cardiomyocyte apoptosis by activating the NIK/IKK/NF-κB pathway.** H9C2 cells were transfected with DANCR shRNA (sh-DANCR) and control (sh-NC), and then treated with OGD and/or B022 (an inhibitor of NIK). (**A**) The expression of DANCR was detected with RT-qPCR. (**B**–**D**) The protein expression of p-RXRA and TRAF2 was detected with Western blotting. (**E**, **F**) The activity of NIK and IKK was measured by ELISA. (**G**–**I**) The protein expression of p-Ik-Bα and p-p65 was detected with Western blotting. (**J**) Cell viability was measured by using CCK-8. (**K**) The activity of caspase-3 was assayed by ELISA. (**L**, **M**) Cell apoptosis was assessed by using Flow cytometry. ^*^P < 0.05, ^**^P < 0.01.

We sought to determine whether DANCR regulated apoptosis of cardiomyocytes through the NIK/IKK/NF-κB signaling pathway and found that silencing DANCR sensibly decreased the levels of DANCR, p-RXRA protein and increased TRAF2 protein in H9C2 cells treated with OGD (Figure 8A–8D). Furthermore, silencing DANCR markedly elevated the activity of NIK and IKK and increased the phosphorylation levels of IkBα and p65 in H9C2 cells treated with OGD, while B022, an inhibitor of NIK, inhibited the activity of NIK and IKK and phosphorylated protein expression of IkBα and p65 (Figure 8E–8I). Meanwhile, we also observed that silencing DANCR inhibited proliferation and promoted apoptosis of H9C2 cells treated with OGD, and silencing DANCR increased Caspase-3 activity in H9C2 cells treated with OGD (Figure 8J–8M). Whereas B022 treatment significantly offset the effects of DANCR silencing on H9C2 cells (Figure 8J–8M). The results demonstrated that silencing DANCR promoted OGD-induced cardiomyocyte apoptosis by activating the NIK/IKK/NF-κB pathway.

## DISCUSSION

Myocardial infarction is one of the major causes of sudden cardiac death worldwide, and there are multiple complex cascades involved in this process, including inflammatory response, fibrosis, myocardial injury and other processes [31, 32]. Recent novel therapeutic strategies are to replace missing or nonfunctional cardiomyocytes with stem cells or to reduce apoptosis of cardiomyocytes [33, 34]. Our approach here has been to target DANCR, and block p-RXRA-mediated TRAF2/NIK/IKK/NF-κB pathway to suppress cardiomyocyte apoptosis and improve AMI in mice.

LncRNA differentiation antagonizing non-protein coding RNA (DANCR) is involved in the progression of multiple diseases. Among them, the report by An et al. [13] showed that DANCR expression in blood of patients with atherosclerosis was markedly upregulated. Zhang et al.’s report [12] showed that DANCR expression was markedly upregulated in blood of atherosclerosis patients and endothelial cells treated with ox-LDL, overexpression of DANCR obviously decreased cell viability, induced apoptosis, and elevated the levels of inflammatory cytokines in ox-LDL-caused endothelial cells. Furthermore, Ruan et al.’s report [14] showed that DANCR expression is increased in OGD/R-induced HL-1 cells. Knockdown of DANCR increased cell viability, but reduced apoptosis in OGD/R-exposed HL-1 cells. Huang et al.’s report [35] showed that DANCR deletion suppressed heart failure by alleviating myocardial hypertrophy and fibrosis. Surprisingly, Qiu et al.’s report [15] showed that DANCR expression was markedly upregulated in hypoxia-induced cardiomyocytes, and overexpression of DANCR enhanced the viability and inhibited apoptosis of cardiomyocytes. Li et al.’s report [36] showed that overexpression of DANCR inhibited apoptosis to protect cardiomyocytes by enhancing autophagy and reducing endoplasmic reticulum stress. Tian et al.’s report [37] showed that DANCR alleviated cardiomyocyte injury in AMI by regulating miRNA-509-5p/KLF pathway. Our study found that DANCR expression was downregulated in both cardiac tissues of AMI mice and OGD-induced cardiomyocytes. Overexpression of DANCR sensibly promoted proliferation, inhibited cell apoptosis and the apoptosis related protein caspase-3 activity, decreased Bax protein levels, and elevated Bcl-2 levels in H9C2 cells treated with OGD. These findings in our study are consistent with the above reports [15, 36, 37] and suggested that high expression of DANCR may alleviate the progression of AMI by inhibiting cardiomyocyte apoptosis.

This study showed that DANCR promoted GSK3β-mediated the phosphorylation of RXRA, but had no effect on the expression of RXRA. Previous reports showed that functional alterations triggered by phosphorylation modification of RXRA are involved in the development of multiple diseases [18, 38]. For example, Santiago et al. [18] found that phosphorylation of RXRA promotes metabolic syndrome by mediating c-Jun NH2-terminal kinase (JNK) expression; Jacob et al. [38] reported that serine 22 phosphorylation of RXRA was upregulated in brown adipocytes. These results are consistent with our research that altered phosphorylation levels of RXRA affect its biological function in disease progression. Moreover, we found that Bex, an activator of RXRA, significantly promoted the proliferation and inhibited the apoptosis in cardiomyocytes induced by OGD. These findings are consistent with previous reports shown that phosphorylated RXRA has an inhibitory effect on diseases such as heart failure and myocardial infarction [19, 20].

Apoptosis is induced via specific death receptors, and tumor necrosis factor receptor 2 (TNFR2) is usually recruited to TNFR1 to regulate cell apoptosis [39, 40]. TRAF2 has a conserved C domain, a coiled N domain, and an N-terminal RING E3 ubiquitin ligase domain. Existing reports have shown that TRAF2 plays a key role in signal transduction of inflammatory response, immune response, cell proliferation, and apoptosis [41]. Furthermore, reports have shown that TRAF2 confers protection against OGD/R injury by mediating mitophagy in cardiomyocytes. Importantly, a recent study indicated TRAF2 plays a key role in death ligands-induced necrotic cell death [41]. It’s shown that TRAF2 deletion inhibited cardiac ischemia-reperfusion injury in mice. However, overexpression of TRAF2 resulted in adverse cardiac remodeling and heart failure in mice [40]. Our study showed that RXRA could bind to TRAF2 through auto-phosphorylation, and inhibit TRAF2 expression to reduce the apoptosis of cardiomyocytes induced by OGD.

It’s reported that N-terminally truncated RXRA (tRXRA) is located in the cytoplasm and interacts with TRAF2 to activate the NF-κB signaling pathway, but full-length RXRA is located in the nucleus and not bind to TRAF2 [22]. In addition to the reported binding between RXRA and TRAF2, we have discovered a new interaction in which phosphorylated RXRA cloud bind to TRAF2. By using Co-IP assay, we showed that RXRA could bind to TRAF2 through auto-phosphorylation. Interestingly, Bex, an activator of RXRA phosphorylation, inhibited the protein expression of TRAF2, but overexpression RXRA had no effect on TRAF2 protein expression. Bex markedly promoted the proliferation and inhibited apoptosis of OGD-induced cardiomyocytes via p-RXRA-mediated TRAF2 downregulation. Whereas overexpression of TRAF2 significantly reversed the protective effect of Bex on cardiomyocytes treated with OGD.

The NF-κB pathway is frequently activated in myocardial infarction [25, 42]. In addition, a report [26] suggested that NIK-IKK complex activated the canonical NF-κB pathway. Our study showed that silencing DANCR markedly promoted the activity of NIK and IKK and increased the phosphorylation levels of IkBα and p65 in H9C2 cells treated with OGD, while B022, an inhibitor of NIK, inhibited the activity of NIK and IKK and reduced phosphorylated protein expression of IkBα and p65. Meanwhile, we also observed that B022 significantly offset the effects of DANCR silencing on cell proliferation and apoptosis. These results demonstrated that DANCR inhibited OGD-induced cardiomyocyte apoptosis by blocking the NIK/IKK/NF-κB pathway, this is consistent with previous reports on the NF-κB pathway [25, 42]. Our study shows for the first time that DANCR inhibited cardiomyocyte apoptosis through blocking p-RXRA-mediated activation of the TRAF2/NIK/IKK/NF-κB pathway, alleviating AMI progression and providing a molecular basis for formulating targeted therapeutic strategies for AMI. However, there may be multiple signaling pathways involved in the process of cardiomyocyte death. We only explored that DANCR inhibits cardiomyocyte apoptosis by regulating the TRAF2/NIK/IKK/NF-03BAB pathway, and the role of other signaling pathways are not clear. In addition, the heart is a complex environment, and the role of DANCR *in vivo* needs to be confirmed by a large number of clinical data, which needs further research in the future.

In summary, we demonstrated that DANCR was downregulated in both AMI mice and OGD-induced cardiomyocytes, and overexpression of DANCR suppressed OGD-induced cardiomyocyte apoptosis by blocking p-RXRA-mediated TRAF2/NIK/IKK/NF-κB pathway, thereby ameliorating AMI in mice. Our findings improve the pathogenesis regulatory network of AIM and provide a new perspective for the treatment of AIM.
